# Diagnostic Modalities of Precancerous and Cancerous Cervical Lesions with Special Emphasis on CD31 Angiogenesis Factor as a Marker

**DOI:** 10.1155/2013/243168

**Published:** 2013-09-17

**Authors:** Bandana Sharma, Neetu Singh, Neena Gupta, Pavika Lal, Shefali Pande, Shashi Chauhan

**Affiliations:** Department of Obstetrics & Gynaecology, GSVM Medical College, Kanpur 208002, India

## Abstract

*Objectives*. To evaluate the role of angiogenesis tumor marker CD31 in the detection of precancerous and cancerous cervical lesions and to compare its efficacy with colposcopy and histopathology. *Materials and Methods*. 230 patients with a suspicious looking cervix and an abnormal Pap smear attending the Outpatient Department of Obstetrics and Gynaecology of GSVM Medical College were subjected to a colposcopic examination. 180 patients with suspected colposcopic findings were subjected to a colposcopic directed biopsy. Biopsy tissues were sent for histopathological examination out of which 50 biopsied samples were sent for immunostaining of CD-31. Statistical analysis was done. *Results*. Comparison of microvessel density (MVD) count by haematoxylin and eosin staining (HE) and immunostaining of CD31 in preinvasive group were 4.012 ± 2.57 and 5.44 ± 2.21, respectively, and in invasive group were 9.18 ± 2.32 and 12.82 ± 4.07, respectively, which showed that MVD was higher by CD31 both in preinvasive and invasive group, and it was statistically significant. *Conclusion*. Angiogenesis is a marker of tumor progression, and CD31 fixes up vessel better as compared to HE, so aggressiveness of the tumor can be better predicted by MVD-CD31 as compared to MVD-HE.

## 1. Introduction

Worldwide cervical cancer is the most common gynaecological malignancy and the third most common frequently diagnosed cancer in female after breast and colorectal cancer. It is the commonest cause of gynaecological cancer death and responsible for about 190,000 deaths every year [1].

Both the incidence and mortality from cervical cancer have been reduced by various systematic screening methods and treatment of premalignant cervical lesions. The introduction of practical cytology by Papanicolaou and Traut in 1943 and its later widespread use into clinical practice represented the major development in cervical cancer preventions further assisted by the use of colposcopy which allows the clinician to visually identify these preinvasive lesions on the cervix, determine their extent, and select a site for biopsy confirmation.

Angiogenesis is a better correlate of malignancy and a requisite for metastasis. Tumor growth is thus controlled by the balance between angiogenic factors (vascular endothelial growth factor, fibroblast growth factor, and platelet derived growth factor) and antiangiogenic factors (thrombospondin, angiostatin, and endostatin). Intensity of tumor angiogenesis is supposed to be reflected by intratumoral microvessel density (MVD), but it is difficult to assess revascularization by normal microscopy; therefore various antibodies members for endothelial cells have been used to demarcate the area of vascularity within the tumor out of which CD-31 has the best documented specificity for endothelial cells. CD-31 is a cluster of differentiation molecule with molecular weight of 130–140 Kilodaltons belonging to the immunoglobuin supergene family. It plays a major role in tissue regeneration and is normally found on endothelial cells, platelets, and on macrophages along with the expression on vascular tumors. 

The importance of screening of carcinoma cervix by CD31 angiogenesis marker is that it may provide a better detection of precancerous and cancerous lesions along with its aggressiveness, therefore, being of great help in prognostication of patients. 

## 2. Materials and Methods

This was a prospective study with total number of 250 patients who attended the Outpatient Department of Obstetrics and Gynaecology of GSVM Medical College, Kanpur, from January 2007 to October 2008, out of which 230 patients with suspicious looking cervix and abnormal pap smear were subjected to colposcopy between the 8th and the 12th days of the menstrual cycle. 180 patients with suspected colposcopic findings were subjected to a colposcopy directed biopsy, and tissues were sent for histopathological examination in the Department of Pathology, GSVM Medical College Kanpur. Biopsy tissues of 50 patients were sent for immunostaining of CD-31 angiogenesis tissue marker using streptavidin biotin immunoperoxidase method at a laboratory in Mumbai. A monoclonal antibody against CD-31 was used as a primary antibody.

Patients were studied according to their sociodemographic characteristics and correlation of cytological, colposcopic, and histological findings was done along with comparison of MVD by haematoxylin and eosin staining (HE) and CD-31 immunostaining. Statistical analysis was applied wherever applicable.

## 3. Observations and Results

Mean age for CIN III and carcinoma was 46.39 ± 5.33 years in our study. 34.42% (21/61) of patients with parity ≥3 had CIN III and carcinoma lesions, whereas in patients with parity ≤2 it was only 3.17% (6/189). 52.2% (94/180) of patients with cervical lesions belonged to a lower socioeconomic status, whereas only 13.3% (24/180) belonged to a higher socioeconomic status. Mean age at first coitus for inflammatory lesion was found to be 24.61 ± 3.37 years and for dysplasia and carcinoma was 17.67 ± 3.86 years (Table 1).

A Pap smear was taken in 250 patients out of which the total number of premalignant and malignant cases (mild to severe dysplasia and carcinoma) detected was 95 (38%). 2.8% was an unsatisfactory cytology due to the presence of a large number of red cells or mucus and drying artifacts. Among 180 patients whose tissues were biopsied, distribution of cases according to histopathological findings was inflammatory (63), CIN-I (60), CIN II (30), CIN III (16), and carcinoma (11). Therefore our study showed that inflammation (35%) followed by CIN-I (33.3%) was the commonest histological finding, when colposcopic directed biopsy was done (Table 2). 

Acetowhite epithelium was the most common colposcopic finding in 100% of cases, and also the grade of dysplasia increases as mosaic and punctuation pattern increases. Atypical vessels were the finding which was present in 100% of the cases of carcinoma and in 12.5% cases of CIN III of the whereas no abnormal vessels were seen in inflammatory or CIN-I lesions (Table 3).

Reid's colposcopic index is based on 4 colposcopic signs that is, colour, margin, vascular pattern, and vascular response [2]. Using Reid's colposcopic index in our study, the sensitivity and the specificity were 100% and 87.3%, respectively (Table 4).

Advanced cervical lesions showed higher MVD count both by HE and CD-31 which was statistically significant that is *P* < 0.0001 (Table 5).

**Figure 1:**
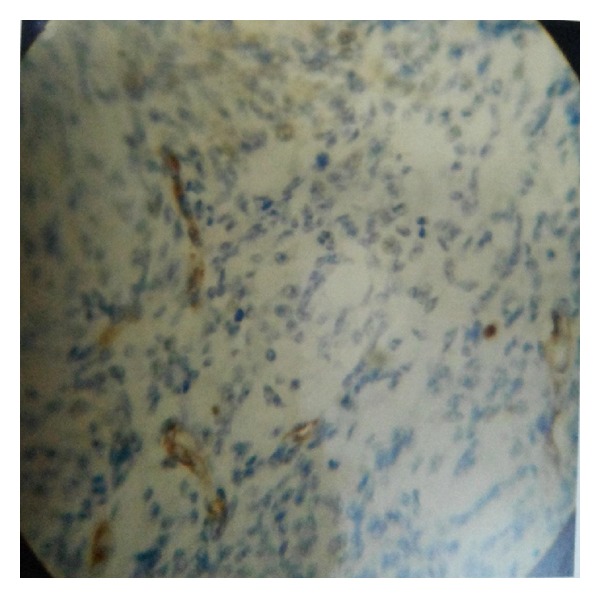
CIN 1 showing low CD31 positivity (×400).

**Figure 2:**
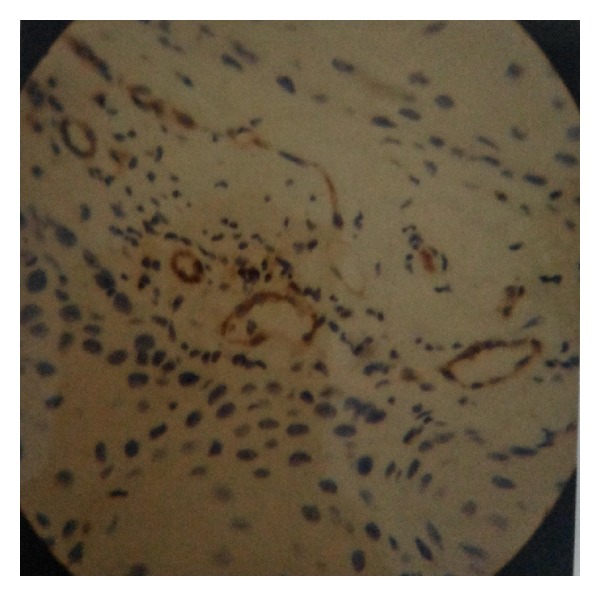
CIN II showing moderate CD31 positivity (×400).

**Figure 3:**
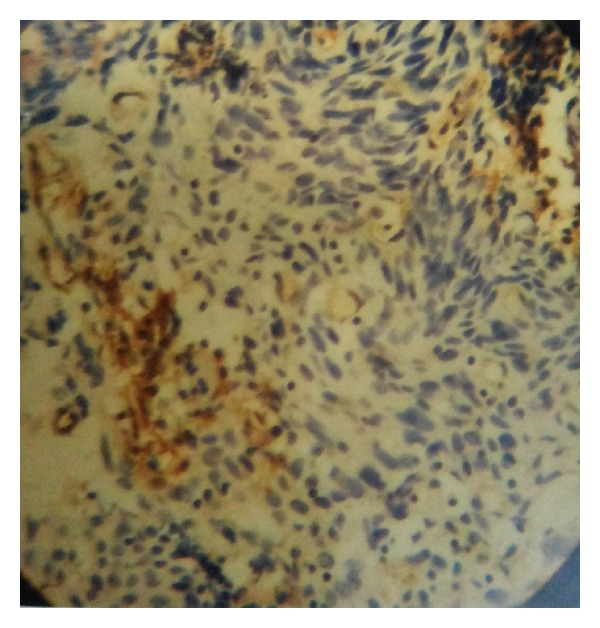
CIN III showing high CD31 positivity (×400).

Mean MVD in preinvasive as well as in invasive lesions by CD-31 immunostaining was higher when compared with HE staining which was also found to be statistically significant (Table 6, Figures 1, 2, and 3).

## 4. Discussion

The detection rate of Pap smear was 38% [3]. In our study, mean age for inflammatory lesions was found to be 28.37 ± 5.74 years whereas that for CIN-I, CIN II, and CIN III was 36.67 ± 7.2, 39.33 ± 6.39, and 46.39 ± 5.33 years, respectively, which was found to be similar in a study conducted by Rohtagi [4]. Severity of lesions increased with increasing parity indicating that trauma and child birth are predisposed to cervical lesions. The results of our study correlate with that of Rohtagi and Wahi et al. [4, 5]. CIN III and carcinoma were found in 52.2% of patients which belonged to a lower socioeconomic status, whereas only 13.3% belonged to higher socioeconomic status which was in concordance with the study conducted by Sankaranarayanan et al. who found that 8% of their cases was a from higher socioeconomic group [6].

In our study, the mean age at the first coitus for inflammatory lesions was found to be 24.67 ± 3.37 years and that for CIN-I, CIN II, CIN III, and carcinoma was 17.67 ± 3.86 years which was similar to the study conducted by Rotkin who found that among patients who began the coitus between the age group of 15 to 17 years had twice the incidence of cervical cancer than the control group [7].

The sensitivity of colposcopic index in our study was 100% as no patients with cervical dysplasia were missed; however, the specificity was 87.3% with no false negatives. Hordhanger et al. reported the accuracy in detecting preinvasive lesions of cervix by colposcopy to 89% as against 99% by colposcopy and targeted biopsy [8].

Ozalp et al. study showed that CIN II and CIN III show higher MVD count as compared to normal and CIN I lesions [9].

Comparison of mean of MVD in the preinvasive and invasive lesions by HE showed that in preinvasive group mean MVD was 4.01 ± 2.57, and in the invasive group it was 9.18 ± 2.32 which shows that MVD was higher in the invasive group as compared to the preinvasive group, and it was statistically significant *P* < 0.0001. Comparison of mean of MVD in the preinvasive and invasive carcinoma by CD31 showed that in preinvasive group mean MVD was 5.44 ± 2.21, and in the invasive group it was 12.82 ± 4.07 showing that MVD was higher in the invasive group, and it was also statistically significant *P* < 0.0001. Dellas et al. also showed similar results [10].

Silva-Filho et al. to evaluated the association between the expression of CD31 in the tumor and histopathologic finding in patients with cancer cervix of the expression; CD31 was significantly associated with tumor size [11].

Comparison of mean MVD count by HE and CD31 shows that mean MVD in the preinvasive group by HE was 4.01 ± 2.57 and by CD31 was 5.44 ± 2.21. It shows that mean MVD detected by CD31 was higher, and it was also statistically significant. Similarly, mean MVD in invasive lesions by HE was 9.18 ± 2.32 and by CD31 was 12.82 ± 4.07. It shows that mean MVD detected by CD31 was higher, and it was also statistically significant. Angiogenesis is a marker of tumor progression, and CD31 fixes up vessel better as compared to HE, so the aggressiveness of the tumor can be assessed better by CD31 as compared to HE.

Sapino et al. also showed increased expression of CD31 by cell of ductal carcinoma in situ and invasive carcinoma of breast which was associated with more poorer prognosis [12].

Arihiro et al. also concluded increased CD 31 expression with increased incidence of lymph node metastasis in breast cancer [13].

In a study conducted by Alexander-Sefre et al., the detection rate for tumor metastasis in lymphovascular space is three fold greater by CD31 marker as compared to conventional HE staining methods [14].

## 5. Conclusion

With the introduction of various effective screening programmes, the mortality and morbidity have declined rapidly associated with cervical cancer but, still there is a need for a better screening test because the existing screening test lacks the appropriate sensitivity and specificity.

The Pap test may be unable to achieve concurrently high sensitivity and specificity [3].

The major drawback of primary colposcopy is its low specificity with the consequence of high false positive rates and over treatment in substantial number of cases [15].

MVD was higher both in the invasive and the preinvasive groups by CD31 immunostaining as compared to HE. We concluded that CD31 immunostaining is a better quantitative prognostic marker, so it should be done in all cases of preinvasive and invasive lesions.

More studies are required for the evaluation of this marker to be incorporated in the existing screening programs because of its limited availability and higher cost.

## Figures and Tables

**Table 1:** Sociodemographic characteristics of the study population.

Histopathological findings	Age (mean ± SD)	Parity (number)				Socioeconomic status (number)			Age at first coitus (number)		
		*P*_0_	*P*_1_	*P*_2_	*P*_3_	Low	Middle	High	10–20 yrs	21–30 yrs	>30 yrs
Normal	27.71 ± 5.44	17	27	23	03	21	36	13	04	64	02
Inflammatory	28.37 ± 5.74	7	28	26	02	27	25	11	03	59	01
CIN-I	36.67 ± 7.20	—	19	21	20	30	21	09	35	25	—
CIN-II	39.33 ± 6.39	—	08	07	15	17	09	04	28	02	—
CIN-III	46.39 ± 5.33	—	03	03	10	11	05	—	16	—	—
Carcinoma		—	—	—	11	09	02	—	11	—	—

**Table 2:** Correlation of cytological findings with histopathological findings in 180 patients.

Cytological finding	Histopathological findings					
	Normal	Inflammatory	CIN-I	CIN-II	CIN-III	Carcinoma
Normal	—	08	05	—	—	—
Inflammatory	—	51	11	02	—	—
Mild dysplasia	—	04	40	04	02	—
Moderate dysplasia	—	—	04	24	03	—
Severe dysplasia	—	—	—	—	11	—
Carcinoma	—	—	—	—	—	11

Total	—	63	60	30	16	11

**Table 3:** Colposcopic findings of the study population (230).

Histological diagnosis	Normal	Colposcopic findings						
		Acetowhite	Mosaic	Punctation	Atypical vessels	Schiller's positive	Unsatisfactory	Total
		No. (%)	No. (%)	No. (%)	No. (%)	No. (%)	No. (%)	
Normal (%)	50	—	—	—	—	—	—	50
Inflammatory (%)	—	63 (100)	06 (9.52)	08 (12.69)	—	14 (22.22)	—	63
CIN-I (%)	—	60 (100)	15 (25)	22 (36.6)	—	36 (60)	02 (3.33)	60
CIN-II (%)	—	30 (100)	14 (46.6)	16 (53.3)	02 (6.67)	19 (63.3)	—	30
CIN-III (%)	—	16 (100)	06 (37.5)	04 (25)	02 (12.5)	16 (100)	06 (37.5)	16
Carcinoma (%)	—	11 (100)	11 (100)	11 (100)	11 (100)	11 (100)	—	11

**Table 4:** Correlation of colposcopic findings using colposcopic index with histopathological findings.

Colposcopic index	Histopathological findings				
	Inflammatory (No.)	CIN-I (No.)	CIN-II (No.)	CIN-III (No.)	Carcinoma (No.)
0	—	—	—	—	—
1	32	17	—	—	—
2	23	23	—	—	—
3	06	12	09	—	—
4	02	08	08	05	—
5	—	—	08	07	—
6	—	—	05	04	—
7	—	—	—	—	—
8	—	—	—	—	11

Total	63	60	30	16	11

**Table 5:** Comparison of mean vascular density in different stages of preinvasive and invasive lesions using haematoxylin and eosin staining and CD-31 immunostaining (per high power field).

S. no.	Stage	No. of cases	MVD-HE		MVD-CD31	
			Mean ± SD	S.E. of mean	Mean ± SD	S.E. of mean
1	CIN-I	17	2.53 ± 1.007	0.24	4.06 ± 0.83	0.20
2	CIN-II	05	3.64 ± 1.10	0.29	5.40 ± 1.84	0.48
3	CIN-III	07	8.43 ± 2.64	0.99	8.86 ± 1.46	0.55
4	Carcinoma	11	9.18 ± 2.32	0.69	12.82 ± 4.07	1.23

**Table 6:** Comparison of mean vessel density by HE and CD-31 staining (per high power field).

S. no.	Group	No. of cases	Mean ± SD	*P* value
Preinvasive				
1	HE	39	4.01 ± 2.57	0.011
2	CD31	39	5.44 ± 2.21	

Invasive				
3	HE	11	9.18 ± 2.32	0.018
4	CD31	11	12.82 ± 4.07	

## References

[1] Dennis S, Nadeem R, Abu Rustam (2011). Cancer of the cervix. *T-Lindes Operative Gynaecology*.

[2] Reid RI, Coppleson M, Apgar B, Brotzman G, Spitzer M (2008). The reid colposcopic index for lesion grading. *Colposcopy: Principles and Practice*.

[3] Fahey MT, Irwig L, Macaskill P (1995). Meta-analysis of pap test accuracy. *American Journal of Epidemiology*.

[4] Rohtagi A (1989). Evaluation of cervix with colposcope, acetowhite patches ic colposcopically screened womens. *The Journal of Obstetrics and Gynaecology of India*.

[5] Wahi PN, Mali S, Luthra UK (1969). Factors influencing cancer of the uterine cervix in North India. *Cancer*.

[6] Sankaranarayanan R, Krishnan Nair M, Jayaprakash PG (1995). Cervical cancer in Kerala: a hospital registry-based study on survival and prognostic factors. *British Journal of Cancer*.

[7] Rotkin ID (1962). Relation of adolescent coitus to cervical cancer risk. * The Journal of the American Medical Association*.

[8] Hordhanger P, Mehra U, Torranga A (1976). Comparison of colposcopy biopsy and cold knife conization in patients with abnormal cytology. *Surgery, Gynecology and Obstetrics*.

[9] Ozalp S, Yalcin OT, Oner U, Tanir HM, Acikalin M, Sarac I (2003). Microvessel density as a prognostic factor in preinvasive and invasive cervical lesions. *European Journal of Gynaecological Oncology*.

[10] Dellas A, Moch H, Schultheiss E (1997). Angiogenesis in cervical neoplasia: microvessel quantitation in precancerous lesions and invasive carcinomas with clinicopathological correlations. *Gynecologic Oncology*.

[11] Silva-Filho AL, Traiman P, Triginelli SA (2006). Association between CD31 expression and histopathologic features in stage IB squamous cell carcinoma of the cervix. *International Journal of Gynecological Cancer*.

[12] Sapino A, Righi L, Cassoni P, Papotti M, Gugliotta P, Bussolati G (2001). Expression of apocrine differentiation markers in neuroendocrine breast carcinomas of aged women. *Modern Pathology*.

[13] Arihiro K, Kaneko M, Fujii S, Inai K (1998). Loss of CD9 with expression of CD31 and VEGF in breast carcinoma, as predictive factors of lymph node metastasis. *Breast Cancer*.

[14] Alexander-Sefre F, Singh N, Ayhan A, Salveson HB, Wilbanks G, Jacobs IJ (2003). Detection of tumour lymphovascular space invasion using dual cytokeratin and CD31 immunohistochemistry. *Journal of Clinical Pathology*.

[15] Pete I, Toth V, Bosze P (1998). The value of colposcopy in screening cervical carcinoma. *European Journal of Gynaecological Oncology*.

